# Pre-Eclampsia: From Etiology and Molecular Mechanisms to Clinical Tools—A Review of the Literature

**DOI:** 10.3390/cimb45080391

**Published:** 2023-07-25

**Authors:** Sara Tabacco, Silvia Ambrosii, Valentina Polsinelli, Ilaria Fantasia, Angela D’Alfonso, Manuela Ludovisi, Sandra Cecconi, Maurizio Guido

**Affiliations:** 1 Unit of Obstetrics and Gynecology, San Salvatore Hospital, 67100 L’Aquila, Italy; 2 Department of Life, Health and Environmental Sciences, University of L’Aquila, 67100 L’Aquila, Italy

**Keywords:** pre-eclampsia, etiology of pre-eclampsia, molecular mechanisms of pre-eclampsia

## Abstract

Pre-eclampsia is a severe pregnancy-related complication that manifests as a syndrome with multisystem involvement and damage. It has significantly grown in frequency during the past 30 years and could be considered as one of the major causes of maternal and fetal morbidity and mortality. However, the specific etiology and molecular mechanisms of pre-eclampsia are still poorly known and could have a variety of causes, such as altered angiogenesis, inflammations, maternal infections, obesity, metabolic disorders, gestational diabetes, and autoimmune diseases. Perhaps the most promising area under investigation is the imbalance of maternal angiogenic factors and its effects on vascular function, though studies in placental oxidative stress and maternal immune response have demonstrated intriguing findings. However, to determine the relative importance of each cause and the impact of actions aiming to significantly reduce the incidence of this illness, more research is needed. Moreover, it is necessary to better understand the etiologies of each subtype of pre-eclampsia as well as the pathophysiology of other major obstetrical syndromes to identify a clinical tool able to recognize patients at risk of pre-eclampsia early.

## 1. Introduction

Pre-eclampsia is a severe pregnancy-related complication characterized by a syndrome with multisystem involvement and damage based on hypertension (systolic blood pressure of 140 mmHg or diastolic blood pressure of 90 mmHg) that first shows up after 20 weeks of gestation.

Pre-eclampsia and hypertension during pregnancy have significantly grown in frequency during the past 30 years [1]. Pre-eclampsia, which has a 3–5% global frequency, is a major cause of maternal and fetal morbidity and mortality as well as large healthcare expenses globally [2]. When pre-eclampsia exhibits severe symptoms of eclampsia or hemolysis, increased liver enzymes, and low platelet (HELLP) syndrome, maternal mortality is even higher. Pre-eclampsia is one of the more prevalent pregnancy problems; however, its specific etiology and pathophysiology are still poorly known and could have a variety of causes. Pregnancy termination is now the only treatment available. Pre-eclampsia, though, can linger after delivery and, in rare instances, come on suddenly during the postpartum period. Pre-eclampsia following childbirth, whether de novo or persistent, has become a significant risk factor for peripartum morbidity. Additionally, mothers with a history of pre-eclampsia and their offspring continue to be at an elevated risk of developing future cardiovascular and metabolic disorders [3]. 

## 2. Etiology and Molecular Mechanisms

### 2.1. Angiogenic Factors

Pre-eclampsia is linked to fetal complications including intrauterine growth restriction and premature birth as well as uterine and placental abnormalities [4]. Trophoblast invasion into the uterine arteries represents the start of placentation in a healthy pregnancy. To supply the fetus with nutrition, arterioles are transformed into high-capacitance and high-flow arteries [5]. Vascular remodeling is disrupted in pre-eclamptic pregnancy due to insufficient trophoblast invasion, which narrows the mother’s blood vessels and causes placental ischemia [5]. Hypoxia, oxidative stress, and the release of antiangiogenic factors such as soluble fms-like tyrosine kinase 1 (sFLT1) might result from inadequate oxygen perfusion within the uterine vasculature [2]. By elevating blood pressure, sFLT1 causes vasoconstriction and aids in maternal metabolic dysfunction [6]. 

A decrease in placental growth factor (PIGF), a member of the vascular endothelial growth factor (VEGF) family, is similarly connected to an increase in sFLT1 [6]. Placental dysfunction and the clinical manifestation of pre-eclampsia are further exacerbated by the imbalance of sFLT1 and PIGF [6]. 

### 2.2. Inflammation

During pregnancy, women experience a physiological inflammatory response, which is a physiological stress response that helps maintain the immune balance at the maternal–fetal interface. This protects the developing fetus from disturbances and does not have an impact on or cause abnormal symptoms in pregnant women [7]. 

Pre-eclampsia-related pregnancy problems can emerge when the inflammatory response is overactivated and pathological inflammation sets in, which can cause immunological dysregulation and vascular endothelial damage [6]. Its progression is linked to the inflammatory condition at the maternal–fetal interface throughout pregnancy, according to an increasing number of studies [8,9]. As a result, women with pre-eclampsia may display an excessive inflammatory response, with a considerable rise in proinflammatory cytokines. This disease can also manifest in early pregnant women in an inflammatory response condition [8], and can develop because of poor or insufficient inflammation resolution, according to numerous research works [10]. Indeed, pre-eclampsia may be prevented by reducing the inflammatory response [9]. Early in pregnancy, trophoblast cell invasion into the uterine wall results in a modification of the uterine spiral arteries. Reactive oxygen species (ROS) generation increases and oxidative stress in the placenta is induced if there is defective invasion or unmodeled arteries at this stage. The production of proinflammatory molecules rises with the induction of oxidative stress, which induces the inflammatory response and causes vascular endothelial dysfunction. Placental injury brought on by hypoxia and reperfusion is consistent with a pathological inflammatory response that can result in endothelial damage and a systemic inflammatory response, both of which can contribute to the development of pre-eclampsia [11,12]. The severity of this inflammatory overreaction is determined by genetic and environmental factors [12].

### 2.3. Inflammatory Cytokines

Reduced uterine perfusion during pregnancy is an important initial event in pre-eclampsia. Inflammatory cytokines are thought to link placental ischemia with cardiovascular and renal dysfunction [13]. In normal pregnancy, Tumor Necrosis Factor (TNF-α) is low in the first trimester and subsequently increases with advancing gestational age [14]. Some studies report higher TNF-α levels in women with established pre-eclampsia [15,16], and increased levels of TNF-α antigen and mRNA have been described in placental tissue from pre-eclamptic women [17]. Because TNF-α may impair insulin signaling, inhibit lipoprotein lipase, induce Plasminogen Activator Inhibitor-1 (PAI-1), and directly contribute to endothelial dysfunction, this cytokine may be involved in the pathogenesis of pre-eclampsia [18]. Macrophages and dendritic cells are the major antigen-presenting cells in the uterus, and they facilitate the adaptation of the immune response to prevent the rejection of an embryo [19]. Several studies have found a statistically significant increase in macrophages and dendritic cells in pre-eclamptic placentas compared to placentas from normotensive pregnancies [19,20]. An increase in the concentration of cytokines, molecules capable of recruiting macrophages, and dendritic cells has also been found in pre-eclamptic placentas [20]. The increased presence of cytokines, macrophages, and dendritic cells in pre-eclamptic placentas supports the hypothesis that an inflammatory milieu presents in women with pre-eclampsia [20].

### 2.4. Maternal Infection

Since the beginning of the 20th century, maternal infection has been linked to the etiology of pre-eclampsia and eclampsia. According to Albert’s theory (“a latent microbic endometritis”), bacteria-induced putrefactive alterations in the uterine cavity are the cause of the “toxins” that cause eclampsia. In fact, “Bacillus eclampsiae” was suggested as the culprit [21,22]. Because pre-eclampsia and eclampsia do not exhibit the normal symptoms of an infectious condition, such as a fever, this viewpoint gradually lost popularity. But only in the last few years has the theory that microbes may contribute to the development of pre-eclampsia and eclampsia appeared in the literature. A link exists between periodontal disease, urinary tract infection, SARS-CoV-2 infection, and maternal gut dysbiosis [23,24].

Studies have linked pre-eclampsia to the microbial colonization of the maternal urinary tract. Urinary tract infections have been linked to pre-eclampsia, according to a systematic study [25]. However, the case definitions have been broad and have grouped asymptomatic bacteriuria, lower urinary tract infections, and pyelonephritis [26,27]. Evidence for the connection with pre-eclampsia either deteriorates or vanishes when subgroup analysis is conducted. Pre-eclampsia cannot be brought on by asymptomatic bacteriuria, which is not accompanied by a systemic inflammatory response [28]. The development of high blood pressure, proteinuria, a low platelet count, and glomerular fibrinogen deposits occurs after intravenous low-dose endotoxin administration in pregnant rats on day 14 of gestation, according to experimental findings supporting a causal relationship between infection, systemic inflammation, and pre-eclampsia [29]. A systemic inflammatory response and the activation of thrombin through the release of tissue factor are induced by bacterial endotoxins. The pathophysiology of pre-eclampsia is thought to be influenced by these pathways.

Early in the COVID-19 pandemic, it was discovered that a subset of nonpregnant patients experienced hypertension, proteinuria, thrombocytopenia, elevated liver enzymes, and low platelet count (HELLP) syndrome. These symptoms are similar to pre-eclampsia and hemolysis. Pre-eclampsia with severe features of eclampsia and HELLP syndrome are all significantly more likely to develop in pregnant women who have SARS-CoV-2 infection [23]. The following onsets of pre-eclampsia and SARS-CoV-2 infection also have a dose–response relationship [24]. Pre-eclampsia risk is five times higher in patients with severe COVID-19 than in those with asymptomatic COVID-19 [24]. Pre-eclampsia typically develops 3.8 weeks after a mother contracts SARS-CoV-2 [30]. One theory suggests that endothelial dysfunction may play a role in the causal relationship between pre-eclampsia and SARS-CoV-2 infection. In fact, SARS-CoV-2 can cause endotheliitis by infecting endothelial cells that ordinarily express the angiotensin-converting enzyme 2 (ACE2), one of the virus’s cell entrance receptors [31,32]. This susceptibility ultimately results in the multisystemic nature of the syndrome, which includes not only renal involvement but also central nervous system dysfunction and seizures. Endothelial infection can trigger the activation of thrombin, intravascular inflammation (i.e., a cytokine storm), and damage to the microvasculature in target organs [33,34]. Interestingly, SARS-CoV-2 infection and the resolution of hypertension in pregnant women with COVID-19 infection who experienced pre-eclampsia-like symptoms occurred without the delivery of the fetus or placenta [35]. In nonpregnant subjects with COVID-19, the levels of sFlt-1, a marker of endothelial dysfunction, were higher in serum and plasma [36]. This result is in line with a study conducted on pregnant women who had severe COVID-19 and had elevated maternal plasma levels of sFlt-1 as well as a high sFlt-1/PlGF ratio [37]. It may be due to genetic vulnerability that some COVID-19-infected women have pre-eclampsia while others do not [38,39].

The microbiota in the human gut is crucial for host nutrition, energy production, and immunological defenses against possible pathogens [40,41]. The necessity to sustain the growth and development of the fetus and placenta causes a significant restructuring of energy distribution (harvesting, storage, and expenditure) throughout a normal pregnancy. Reported for the third trimester of pregnancy, gut microbiota remodeling during a typical human pregnancy showed an overall rise in Proteobacteria and Actinobacteria and a decrease in microbial richness [42]. When given to germ-free mice, the third-trimester intestinal contents of healthy pregnant women enhanced obesity and insulin resistance [43], which have been linked to the proinflammatory properties of the gut microbiota [42].

Gut dysbiosis, or an imbalance in the microbial populations of the human gut, is currently thought to contribute to the onset of atherosclerosis, hypertension, proteinuria, cardiometabolic syndrome [44,45], and more recently, pre-eclampsia [46,47]. It has been determined that the transplantation of fecal material from hypertensive, nonpregnant, human people to germ-free mice causes hypertension, establishing a causal relationship between gut dysbiosis and cardiovascular disease [48]. Similarly, susceptible mice (such as apolipoprotein E-null mice) can contract atherosclerosis through transplanted feces from susceptible mice [49]. Trimethylamine-N-oxide (TMAO), a metabolite produced by bacteria from choline and carnitine that is abundant in gut dysbiosis and has been proven to hasten atherosclerosis formation, has been credited, at least in part, for this impact [49,50].

Changes in the human gut flora have been linked to pre-eclampsia, which lasts until six weeks after delivery [51]. Firmicutes, Clostridia, Clostridiales, and Ruminococcus are less prevalent, whereas Bacteroidetes, Proteobacteria, Actinobacteria, Bacteroidia, Gammaproteobacteria, and Enterobacteriaceae are more prevalent [52]. Furthermore, that study offered proof of causation: pre-eclampsia syndrome was developed in mice after receiving fecal transplants from pregnant women with the condition [53]. The experimental protocol entailed giving nonpregnant mice treated with antibiotics for five days (to modify gut flora) and kept in pathogen-free environments feces from pregnant women who developed pre-eclampsia and those who did not [53]. The mice were mated six weeks following the initial fecal infection [53]. 

Pregnant mice who received fecal microbiota transplants from mothers with pre-eclampsia experienced high systolic blood pressure, proteinuria, and fewer live pups than mice who got the transplants from mothers who had a normotensive pregnancy [53]. All the data point to the involvement of gut dysbiosis in pre-eclampsia. However, more research is required to determine the precise mechanisms that could explain this phenomenon as well as why some pregnant women are more susceptible than others.

### 2.5. Obesity and Metabolic Disorders

Obesity worsens metabolic abnormalities such as insulin resistance, inflammation, and atherosclerosis disease, which raises the risk for PE in comparison to a normal-weight person [10]. Understanding if and how the reduction in maternal obesity can ameliorate pre-eclampsia and its aftereffects is urgently needed because both the incidence of maternal obesity and cases of pre-eclampsia are increasing globally [54]. 

Adipocytes, immune cells, and stromal vascular cells make up adipose tissue, which functions as an endocrine organ that plays a role in regulating systemic inflammatory and immunological responses [11]. Adipokines such as tumor necrosis factor alpha (TNF-α) and interleukin-6 (IL-6) are released from adipose tissue macrophages and other immune cells and increase the cell-to-cell transmission of local and systemic inflammation, respectively, from central or visceral adipose tissue depots [55]. The modification of the decidual vasculature and invasion of trophoblasts at the maternal–fetal interface are crucial for pregnancy success [56]. Due to high levels of circulating proinflammatory immune cells, it is postulated that in pregnancies impacted by obesity, crosstalk between maternal adipose tissue and the maternal–fetal interface may contribute to an inappropriate vascularization of the placenta [12,56]. TNF-α and IL-6, among other inflammatory cytokines, are released. The development of pre-eclampsia is frequently accompanied by metabolic abnormalities in obese women, such as elevated levels of circulating leptin, glucose, insulin, and cholesterol [57]. By controlling brain satiety, temperature control, and glucose metabolism, the pleiotropic hormone leptin contributes to energy homeostasis [58]. Leptin levels rise regularly after eating; however, in obese people, levels rise over the normal range [59]. Leptin is a cytokine that primarily promotes inflammatory conditions, with both innate and adaptive immune responses [60]. The development of autoimmune disease, as well as reproductive and gestational abnormalities, has been linked to low-grade inflammation and hyperleptinemia in obese individuals [59,60]. Leptin is primarily obtained from two sources during pregnancy: adipose tissue and the placenta. Leptin controls systemic inflammatory responses and vascular function during a healthy pregnancy, both of which influence placentation [59]. Additionally, cholesterol may influence how a pregnancy turns out [10]. The two sources of circulating cholesterol are food intake and cellular production. Although cholesterol is also stored in adipose tissue [61], the liver is largely responsible for maintaining cellular cholesterol production and homeostasis [62]. According to recent research, adipose tissue is a significant location for the storage of free cholesterol, and a rise in cholesterol accumulation is correlated with obesity [63]. About 50% of the body’s cholesterol is kept in adipose tissue in an obese state [63]. In addition to its structural role in the plasma membrane, where it provides cellular support, cholesterol also plays a crucial role in cell signaling [64]. Another factor in the atherogenic process is the proportion of the molecules that carry cholesterol, low-density lipoproteins (LDLs), to high-density lipoproteins (HDLs). The development of plaque, atherosclerotic disease, and subsequent systemic inflammation can all be caused by elevated levels of LDLs, the molecules that carry cholesterol from the liver to peripheral tissue [65]. Additionally, cholesterol has been demonstrated to enhance adipose tissue inflammation and remodeling [63]. A typical physiological response to check is an increase in maternal cholesterol during pregnancy, often known as maternal physiological hypercholesterolemia (MPH). 

### 2.6. Gestational Diabetes Mellitus

Gestational diabetes mellitus could be considered as an independent risk factor for pre-eclampsia [65,66]. In fact, it has been demonstrated that women with gestational diabetes have a higher chance of developing pre-eclampsia, according to a retrospective study including 647 pregnancies [65]. Moreover, a systematic review found that having diabetes mellitus before becoming pregnant was connected to an increased risk of developing pre-eclampsia. [67]. Pre-eclampsia with a late onset is thought to have a stronger association with diabetes mellitus than one with an early onset [68]. According to the Hyperglycemia and Adverse Pregnancy Outcome (HAPO) Study Cooperative Research Group, there is a substantial link between maternal hyperglycemia and adverse pregnancy outcomes.

The finding that the risk of pre-eclampsia is decreased when gestational diabetes mellitus is treated with diet, insulin, and metformin [69,70] reinforces the existence of a causal relationship. Insulin therapy for prenatal diabetes mellitus lowers the incidence of pre-eclampsia, according to two randomized clinical trials. Moreover, metformin has been shown to prolong gestation in women with preterm pre-eclampsia (median, 18 days) and reduce the risk of pre-eclampsia [71,72]. Based on the findings of a comprehensive review and meta-analysis, prenatal exercise has also been shown to reduce the rate of gestational diabetes mellitus by 38% and pre-eclampsia by 41% [73].

Given the consistency of the link, its magnitude, the temporal relationship, and the effectiveness of therapies such as insulin and metformin in reducing the rate of pre-eclampsia, the data collectively point to a causal relationship between these two pathological conditions.

### 2.7. Fetal Disease

The following prenatal conditions are specifically linked to the onset of pre-eclampsia: (1) Ballantyne or mirror syndrome [74,75], (2) trisomy 13 or triploidy [76,77], (3) a particular problem associated with multiple gestations (such as twin-to-twin transfusion syndrome or selective fetal growth restriction) [78]. 

Ballantyne syndrome, also known as triple edema, is characterized by the simultaneous edematous state of the mother, fetus, and placenta [79,80]. Fetal hydrops brought on by rhesus immunization [79], CMV [81], parvovirus 19 infections [82], Ebstein’s anomaly [83], aneurysms of the vein of Galen [84], fetal supraventricular tachycardia [85,86], and placental chorioangioma have all been linked to this syndrome [87]. Trisomy 13 or triploidy is another form of pre-eclampsia with fetal illness [76,77,88]. Pre-eclampsia was much more common in twenty-five trisomy 13 cases than in the normal karyotype control (44% vs. 8%, *p* = 0.001), according to a case–control study [89]. When compared to euploid and trisomy 21 pregnancies, women carrying trisomy 13 have greater serum levels of the sFlt-1/PlGF ratio [90], and their placentas show higher levels of sFlt-1 staining [91]. Interestingly, the presence of sFlt-1 on chromosome 13 raises the idea that there is a second copy of chromosome 13.

Pre-eclampsia can also be influenced by multiple pregnancies with twin-to-twin transfusion syndrome or selective fetal growth restriction [78]. The selective termination of pregnancy [92,93] or the loss of one twin during fetal development are typically followed by the resolution of hypertension and proteinuria [94]. The selective termination or fetal death of one twin has also been shown to improve angiogenic and anti-antiangiogenic profiles [95,96]. Together, these findings show that pre-eclampsia can be treated without delivery and fetal impairment may occasionally cause the syndrome to develop. 

### 2.8. Autoimmune Diseases

Pre-eclampsia is not traditionally considered an autoimmune disorder. However, patients with systemic autoimmune diseases, such as systemic lupus erythematosus (SLE) or antiphospholipid syndrome (APS), are at an increased risk for pre-eclampsia [97,98]. The autoimmune mechanisms of disease have been investigated for a number of decades [99,100,101,102,103], and numerous studies have demonstrated that some particular autoantibodies, such as anti-beta-2 glycoprotein-I (ab2GPI), anticardiolipin antibodies (aCL), or lupus anticoagulant (LA), are related to pre-eclampsia [104,105,106,107]. 

However, antibodies against the angiotensin II receptor serve as the most convincing argument for the involvement of an autoimmune process. In a study published over 20 years ago, Wallukat et al. [108] found that pre-eclampsia patients had antibodies that bind to the AT1-AA subtype of the angiotensin II receptor. Since then, a large quantity of data has emerged to suggest that AT1-AA plays a part in the pathophysiology of pre-eclampsia. These findings comprise this evidence: (1) at the time of diagnosis, maternal blood AT1-AA concentrations are higher in 80% of pre-eclamptic women [109]; (2) the amount of AT1-AA in a mother’s serum relates to how severe her hypertension and proteinuria are [110]; (3) giving pregnant rats AT1-AA (either purified AT1-AA from pre-eclampsia patients or endogenous AT1-AA produced in response to the transgenic expression of human renin and angiotensinogen) caused hypertension, proteinuria, and glomerular capillary endotheliosis.

The concentration of serum AT1-AA has been demonstrated to rise following placental ischemia following a decrease in uterine perfusion pressure [111]. Additionally, the inhibition of AT1-AA through the delivery of the epitope-binding peptide “n7AAc” can lower maternal blood pressure and plasma concentrations of the oxidative stress markers endothelin-1, sFlt-1, and isoprostanes [112]. Additionally, the inflammatory mediators TNF [113,114], IL-6 [113,114], and IL-17 [115] can cause pre-eclampsia and increase AT1-AA activity in pregnant rats, which is reversed by an AT1-AA blocker [112].

## 3. Clinical Tools

Interest in pre-eclampsia prediction with clinical tools and biomarkers has recently increased. A successful predictive test would make early diagnosis, focused surveillance, and prompt delivery possible. Using low-dose prophylaxis with aspirin, a biomarker that can identify high-risk pregnant women in the early stages of pregnancy (less than 16 weeks), to reduce preterm illness has clinical relevance in reducing preterm pre-eclampsia (and associated premature birth and perinatal morbidity) [116]. The PHOENIX trial provides conclusive evidence of the value of identifying patients with higher pre-eclampsia risk in late pregnancy (allowing additional surveillance and well-timed delivery) [117]. Strong proof that planned delivery for pre-eclamptic patients lowers maternal morbidity when compared to expectant care was presented by this randomized study [117]. 

The greatest clinical benefit will likely come from novel screening tests that identify women who are at a high risk of contracting a disease or who already have one, allowing for risk stratification for ongoing therapy. Such diagnostics in pre-eclampsia may be able to identify women who might benefit from closer clinical supervision and delayed delivery. On the other hand, they might spot individuals with minimal risk who could safely cut back on antenatal visits. Pre-eclampsia screening assays typically perform better closer to the onset of the disease [118], i.e., prospective biomarkers that predict term pre-eclampsia perform better when sampled in later gestations as opposed to early ones.

Blood pressure readings have been used for more than a century as a pre-eclampsia-screening tool. However, hypertension is typically only helpful when pre-eclampsia has already begun to develop, and it is only marginally effective at predicting pre-eclampsia in the future. The identification of clinical risk variables for pre-eclampsia [119,120] is another screening test that is currently used in early pregnancy, but it again has extremely poor predictive power. 

Two screening assays that have been developed over the past ten years are now used in some clinical settings. The first is a screening test conducted in the first trimester that identifies those who are at an increased risk of preterm pre-eclampsia. The second is intended for use in the later stages of pregnancy when it is clinically unknown if pre-eclampsia is present or likely to develop. This later test is moderately accurate in determining whether pre-eclampsia will develop (positive predictive value) but very accurate in excluding the development of pre-eclampsia within the next week. 

Today, pre-eclampsia risk can be classified based on maternal features and pregnancy-related factors. Several recommendations list the following conditions as “high”-risk factors for pre-eclampsia: prior pre-eclampsia, chronic renal disease, chronic hypertension, prior diabetes mellitus, and autoimmune disease (particularly antiphospholipid syndrome) [119,120]. Other “moderate” risk factors mentioned in some guidelines include multifetal gestation, nulliparity, advanced age and high body mass index, an interpregnancy interval of more than 10 years and family history of pre-eclampsia, the use of assisted reproductive technology, and maternal age [119,120].

The benefit of using these recommendations is that they may be applied easily and without cost or further testing to all pregnant women. Unfortunately, they perform poorly in terms of sensitivity and predictive performance for late-gestation pre-eclampsia. In fact, according to the NICE (The National Institute for Health and Care Excellence) recommendations, only 41% of women who are going to give birth prematurely will have a screen-positive result [121,122].

## 4. First Trimester Screening for Pre-Eclampsia Risk

Over the years, clinicians created a new first trimester screening algorithm based on numerous characteristics to help identify women at risk of pre-eclampsia early. This method consists of both laboratory and clinical techniques. It combines levels of circulating Placental Growth Factor (PlGF), Doppler ultrasonography measurements of maternal uterine artery resistance, and mean arterial blood pressure. Compared to clinical risk factors alone, this test is more accurate at predicting preterm pre-eclampsia. It successfully diagnoses 82% of instances, which is twice as many as when clinical criteria are applied in accordance with NICE guidelines [123]. It dramatically lowers preterm pre-eclampsia when used to determine who should be provided aspirin to avoid pre-eclampsia. As a result, there are fewer hospital expenses and less long-term financial and human consequences related to preterm birth [124,125]. 

The algorithm-based screening method developed by the Fetal Medicine Foundation, which incorporates maternal variables, mean arterial pressure, uterine artery pulsatility index, and PlGF, outperforms the screening techniques currently advised by the National Institute for Health and Care Excellence and ACOG [125]. The ASPRE study (Aspirin for Evidence-Based Pre-eclampsia Prevention) used a similar screening methodology with the addition of maternal serum PAPP-A (pregnancy-associated plasma protein-A) to identify women in the first trimester who were at a high risk of pre-eclampsia [126]. Then, until 36 weeks gestation, high-risk women were randomly assigned to receive either 150 mg of aspirin or a placebo daily. The detection rate of preterm pre-eclampsia was 76.7% [127,128]—and 43.1% for term pre-eclampsia—with a false-positive rate of 9.1%. Daily low-dose aspirin use in high-risk women was associated with a significantly lower incidence of preterm pre-eclampsia than placebo. These findings imply that early illness detection using imaging investigations and plasma biomarker screening enables early treatment and potential disease prevention.

## 5. Potential Biomarkers

For clinicians, the challenge of recent years has been to identify potential biomarkers that can be used to predict pre-eclampsia early and identify the molecular mechanisms of pre-eclampsia pathogenesis. 

It is currently believed that the number of Tregs (regulatory T-cells) in the peripheral blood has the potential to be a biomarker for assessing the risk of pre-eclampsia, thus facilitating the monitoring of patients with a high risk of pregnancy. However, these proposals are still in the research phase and require further systematic evaluation in more clinical trials. Tregs change throughout pregnancy and reach a maximum in local and systemic Treg expansion in midpregnancy [129], followed by a decrease in their proportion [130]. A recent meta-analysis by Green et al. showed that healthy pregnant women have a significantly higher number of Tregs in the peripheral blood compared to pregnant women with pre-eclampsia. Moreover, healthy pregnant women have a higher number of Tregs in the maternal meconium compared to pregnant women with pre-eclampsia, but there is no significant difference. It is speculated that this result may arise from the poor phenotype of the decidual tissue [131]. Pre-eclampsia pathogenesis is currently considered to be associated with the placental immune system. However, the specific impact of these immune cell differences on pre-eclampsia needs to be further investigated.

Moreover, in recent years, several studies have speculated the role of inflammatory genes in pre-eclampsia. Placental RNA could be released into the maternal circulatory system during early gestation, and maternal plasma mRNA profiles correlate with placental gene expression profiles [132]. A large number of placenta-specific genes have been identified in maternal plasma in pre-eclampsia, and a measurement of circulating placental RNA (cpRNA) may provide an opportunity to predict maternal and fetal complications due to placental dysfunction in late pregnancy [133]. In particular, a recent study by Wang et al. analyzed differential expression genes (DEGs) in pre-eclampsia and non-pre-eclampsia groups, cross-linking with extracted inflammatory-response-related genes to obtain differentially expressed inflammation-related genes (DINRGs). Among the 69 DINRGs associated with the screened pre-eclampsia patients, INHBA, OPRK1, and TPBG were indicated as three key inflammation-associated genes that could be used as potential genetic biomarkers for pre-eclampsia prediction and treatment, and a nomogram was established as a predictive model [134]. 

## 6. Conclusions and Future Directions

Pre-eclampsia is one of the most important causes of maternal morbidity and mortality worldwide. The only definitive treatment—delivery of the fetus and placenta—carries significant consequences for the neonate in terms of morbidity and mortality. In recent decades, the mortality of pre-eclampsia and eclampsia has decreased significantly thanks to increased antenatal maternal surveillance and early interventions. However, the postpartum and lifelong sequelae of pre-eclampsia have risen in number and significance. In fact, women with a history of pre-eclampsia are at increased risk for cardiovascular diseases [135] and dementia [136] later in life. For these reasons, an increased focus on interventional studies during the postpartum asymptomatic phase is necessary to minimize the risk of cardiovascular disease and its potentially fatal complications.

The specific etiology and pathophysiology of pre-eclampsia are still poorly known and could have a variety of causes. To determine the relative importance of each cause and the impact of actions to stop this illness, more research is needed. One of the most promising areas under investigation is the maternal angiogenic factor imbalance and its effects on vascular function, though studies on placental oxidative stress and the maternal immune response demonstrate intriguing findings. In fact, thanks to ongoing development, methods of risk stratification using ratios of proangiogenic factors—such as PlGF—and antiangiogenic factors—such as sFLT1 and sENG—have high detection rates for preterm pre-eclampsia when incorporated into algorithms with other predictive elements [137] and have shown a high negative predictive value as an isolated assay. Furthermore, as reported by a recent meta-analysis by Armaly et al. [138], sFlt-1 and sENG can not only be considered as biomarkers but also responsible for the angiogenic imbalance and generalized endothelial dysfunction characterizing pre-eclampsia. More recently, Simsek et al. also hypothesized a role of expressional regulation and the contraction response of endothelin-1 in the molecular mechanisms of pre-eclampsia pathogenesis [139].
